# Supplementary material for: A UMLS-based spell checker for natural language processing in vaccine safety
Source: BMC Med Inform Decis Mak. 2007 Feb 12;7:3. doi: 10.1186/1472-6947-7-3 (PMC1805499; doi:10.1186/1472-6947-7-3)
Supplement: Additional file 13 — RAP application directory. Sets up directory for RAP (RDF application for PHP) source code [file 1472-6947-7-3-S13.gz › rap/doc/phpdoc/classtrees_resModel.html]

xml version="1.0" encoding="iso-8859-1"?


## Root class ResIterator

- ResIterator

## Root class ResLiteral

- Literal **(Different package)**
  - ResLiteral

## Root class ResModel

- ResModel
  - OntModel

## Root class ResResource

- Resource **(Different package)**
  - ResResource
    - OntResource
      - Individual
      - OntClass
      - OntProperty
  - ResContainer
    - ResAlt
    - ResBag
    - ResSeq
- ResList
- ResProperty

Documentation generated on Thu, 7 Jul 2005 13:41:08 +0200 by phpDocumentor 1.3.0RC3
